# MR imaging of venous malformations: sciatic nerve infiltration patterns and involved muscle groups

**DOI:** 10.1038/s41598-020-71595-6

**Published:** 2020-09-03

**Authors:** Constantin Goldann, Maximilian Helm, Wibke Uller, Claudia Fellner, Simone Hammer, Andreas Deistung, Alexander Gussew, Jonas Rosendahl, Moritz Wildgruber, Walther A. Wohlgemuth, Richard Brill

**Affiliations:** 1 Department of Radiology and Policlinic of Radiology, University Hospital Halle (Saale), Ernst-Grube-Straße 40, 06120 Halle (Saale), Germany; 2 Department of Radiology, University Hospital Regensburg, 93042 Regensburg, Germany; 3 Department of Gastroenterology, University Hospital Halle (Saale), 06120 Halle (Saale), Germany; 4 Department of Radiology, University Hospital Ludwig-Maximilians-Universität, Campus Großhadern, 81377 Munich, Germany

**Keywords:** Peripheral nervous system, Muscle, Circulation, Medical imaging, Magnetic resonance imaging, Paediatrics, Disability, Pain

## Abstract

The aim of this retrospective cross-sectional study was to provide an MRI-based examination framework of venous malformations (VMs) infiltrating the sciatic nerve and determine the frequency of nerve infiltration patterns and muscle involvement in correlation to the patients’ quality of life. Pelvic and lower limb MR images of 378 patients with vascular malformations were examined retrospectively. Pain levels and restriction of motion were evaluated with a questionnaire. Cross-sectional areas of affected nerves were compared at standardized anatomical landmarks. Intraneural infiltration patterns and involvement of muscles surrounding the sciatic nerve were documented. Sciatic nerve infiltration occurred in 23/299 patients (7.7%) with VM. In all cases (23/23; 100%), gluteal or hamstring muscles surrounding the nerve were affected by the VM. Infiltrated nerves were enlarged and showed signal alterations (T2-hyperintensity) compared to the unaffected side. Enlarged nerve cross-sectional areas were associated with elevated pain levels. Three nerve infiltration patterns were observed: subepineurial (12/23; 52.2%), subparaneurial (6/23; 26.1%) and combined (5/23; 21.7%) infiltration. This study provides a clinically relevant assessment for sciatic nerve infiltration patterns and muscle involvement of VMs, while suggesting that VMs in gluteal and hamstring muscles require closer investigation of the sciatic nerve by the radiologist.

## Introduction

Vascular anomalies of the lower limb are an uncommon diagnosis, which begins with an early onset in childhood or adolescence. These anomalies are congenital, grow at the same rate as the child and do not regress over time^1^. The diagnosis is primarily made based upon physical examination and the patient’s history of malformations with subcutaneous parts. However, imaging with ultrasonography and magnetic resonance imaging (MRI) plays an important role in confirming the diagnosis, as well as evaluating the size and extent of the malformation, because physical examinations tend to underestimate these factors^2^. Deeply seated malformations without subcutaneous portions may only be detectable in MR images.

In 1982, a classification was introduced by Mulliken et al. to distinguish hemangiomas from vascular malformations with reference to histological features and findings during the physical examination and is still prevalent today^1^. Based on their work, the International Society for the Study of Vascular Anomalies (ISSVA) established a standardized nomenclature comprising all vascular anomalies and tumors. The classification was last updated in 2018^3^. Until now, a large number of musculoskeletal radiologists do not use the ISSVA classification in clinical practice^4^. According to the ISSVA classification^5^, low-flow vascular malformations incorporate simple venous and combined malformations of venous and lymphatic type, with or without capillary components.

Venous malformations (VMs) represent a large group within vascular malformations^6^, especially in the extremities: In a study of 5,621 patients, 36.8% of all vascular anomalies were venous malformations. 48.3% of venous and 63.3% of combined venous-lymphatic malformations occurred in the extremities^7^.

A VM in the lower limb can infiltrate the sciatic nerve, resulting in sciatic neuropathy with subsequent leg pain and restrictions in motion. Previous research has predominantly consisted of case reports^8,9^. For the current study, we conducted a systematic approach to identify and characterize VM which infiltrate the sciatic nerve and its surrounding muscles, as well as investigated the applicability of a radiological classification system for intraneural vascular anomalies proposed by Prasad et al.^9^ in our group of patients.

## Materials and methods

Approval by the Regensburg University Institutional Review Board (IRB) was obtained (ethics vote number 18-886-104). Written informed consent was waived by the IRB due to the retrospective and non-invasive nature of the study. The study was conducted in accordance with the Declaration of Helsinki. The anonymization of patient data in the research process ensured data protection in accordance with the European General Data Protection Regulation.

The authors declare that this work has not received any funding before or during research. The publication was financially supported by the German Research Foundation (DFG) Open Access Publishing funding programme. There are no relationships to any companies whose products or services may be related to the subject matter of the article.

### Study participants

**Figure 1:**
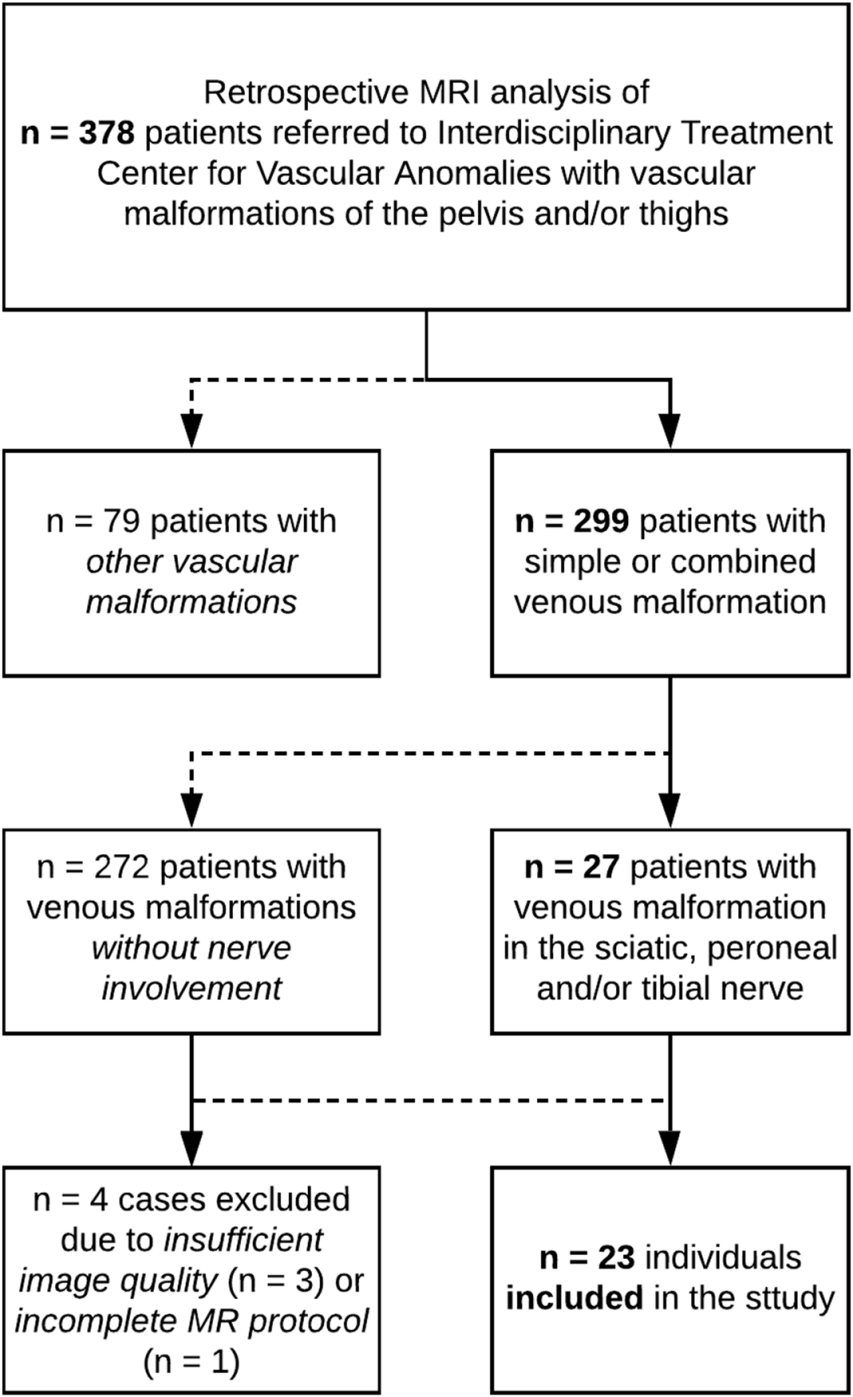
Research flowchart of this study.

We retrospectively analyzed the MRI examinations and clinical records of 378 patients with vascular malformations of the pelvis and thighs. The data was obtained over a 6-year time period (2011 to 2017) at the patients’ initial referral to an interdisciplinary treatment center for vascular anomalies. Inclusion criteria were the presence of a simple or combined VM in the sciatic, peroneal and/or tibial nerve visible in MRI. In case of reduced image quality due to motion artefacts, patients were excluded if the sciatic nerve was not discernable at all predefined landmarks and therefore the nerve’s diameter could not be measured. Additionally, patients with incomplete image acquisition (e.g. missing MR sequences, see section “Image Acquisition”) were excluded. The application of the above-mentioned criteria is shown in the research flowchart (Fig. 1).

### Quality of life

In order to record patient symptoms, in particular, pain and restriction of motion, we had the patients complete a standardized disease-specific questionnaire. Pain intensity was reported on a 0-to-10 Visual Analog Scale. Restriction of motion was investigated using five-point Likert scales in order to quantify the patients’ ability to perform demanding tasks like climbing stairs as well as common daily activities. According to the statements, their impairment was categorized as “no”, “mild” or “severe” restriction in movement.

### Image acquisition

All MR images were acquired using a 3 T MR scanner (Magnetom Skyra, SIEMENS Healthineers, Erlangen, Germany). The standardized imaging protocol contained the following sequences: T2-weighted Short Tau Inversion Recovery (STIR) in the axial plane, T2-weighted turbo-spin echo (TSE) in the axial plane, T2-weighted STIR in the coronal plane, T1-weighted turbo-spin echo in the coronal plane and 3D time-resolved MR angiography with interleaved stochastic trajectories (TWIST) before injection of contrast agent, T1-weighted high-resolution 3D gradient echo with spectral fat saturation (volumetric interpolated breath hold examination, VIBE) after injection of Gadobutrol (Gadovist, Bayer, Leverkusen, Germany) adjusted to the patient’s body weight.

### Analysis

**Figure 2:**
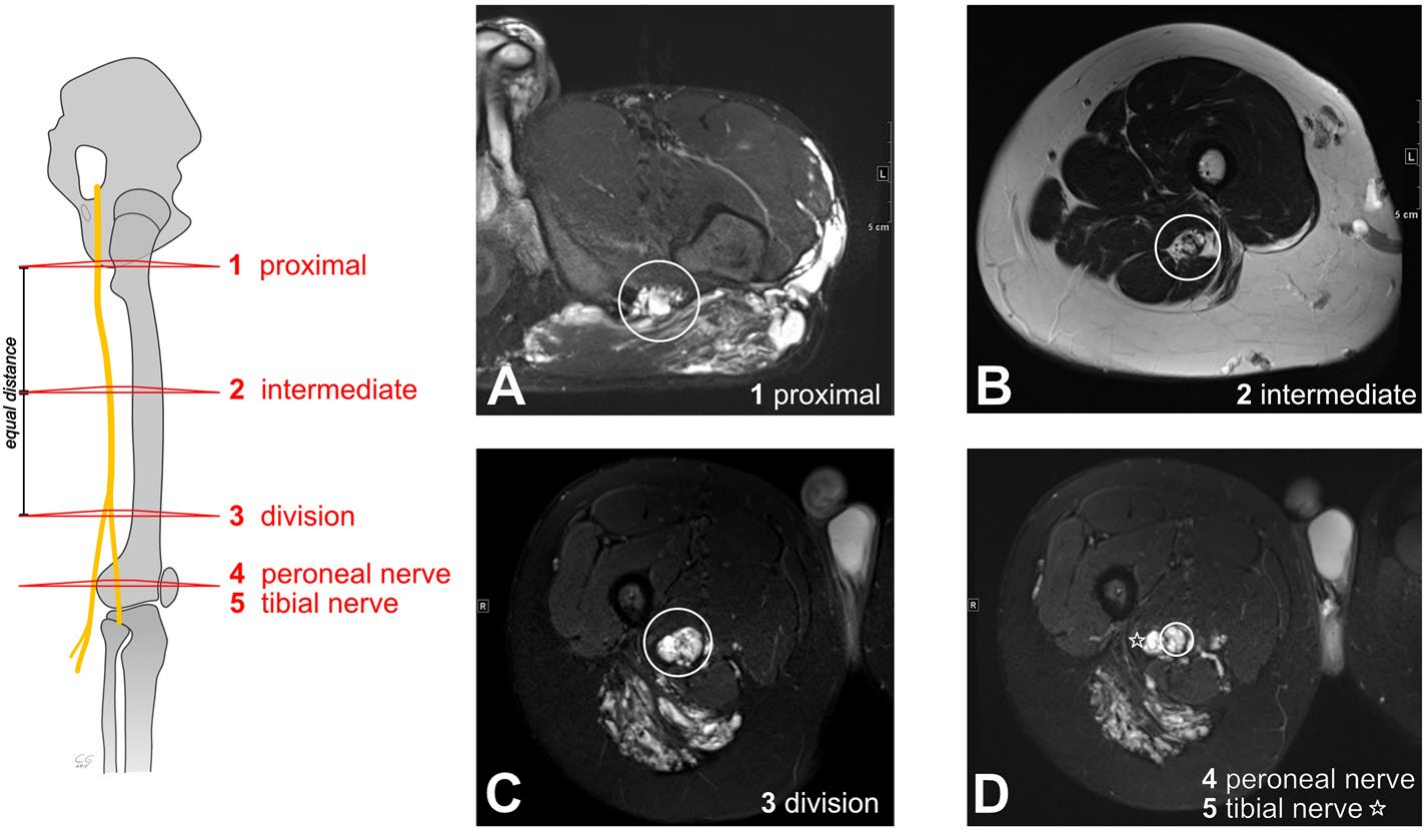
Illustration of the landmarks 1–5 used for measuring nerve cross-section areas and examples of corresponding MR images. All images show venous vascular anomalies with a subepineurial nerve involvement pattern. (**A**) Axial T2 STIR: Sciatic nerve at landmark 1. (**B**) Axial T2 TSE: Sciatic nerve at landmark 2. (**C**) axial T2 STIR: Sciatic nerve at landmark 3 (division into tibial and peroneal nerve). (**D**) axial T2 STIR: Peroneal (landmark 4) and tibial nerve (landmark 5) distal to their division. The peroneal nerve is highlighted with a circle, the tibial nerve with an asterisk.

The author and two radiologists experienced in the field of vascular anomalies interpreted the anonymized MR images in consensus. The extent of the VM itself as well as the infiltrated muscles were documented. To assess morphological differences of the sciatic nerve among affected and healthy limbs, the cross-sectional areas of the nerves were measured in the axial plane. To address anatomical variations of the sciatic nerve, all cross-sectional areas were determined for both thighs at predefined anatomic locations, as illustrated in Fig. 2:

1. proximal landmark at the ischial tuberosity,
2. intermediate landmark in the sectional image of equal distance between landmark 1 and 3,
3. landmark at the division of the sciatic nerve (into tibial and peroneal nerve),
4. proximal tibial nerve, and
5. proximal peroneal nerve.

We also used this nomenclature of landmarks to describe the extent of the sciatic nerve’s involvement in the vascular anomaly. The nerves’ cross-sectional area, $A$, was approximated with an ellipse formula incorporating two nerve diameter measurements: $A=\pi \times \frac{{d}_{1}}{2} \times \frac{{d}_{2}}{2}$ . The major axis of the ellipse is defined as ${d}_{1}$, representing the maximal nerve diameter. The minor axis, ${d}_{2}$, is the second measurement orthogonal to ${d}_{1}$. To ensure inter-individual comparability of patients with unilateral anomalies, ratios between the cross-sectional nerve areas of the affected and healthy thigh, $q$, were computed and considered in the analyses:

$$q=\frac{{A}_{affected}}{{A}_{unaffected}}.$$

In affected areas within the sciatic nerve, the presence of a VM was confirmed on the basis of the following MRI criteria. VMs show intermediate heterogenous signals in T1-weighted sequences and high signals in STIR and T2 sequences. The absence of flow voids is characteristic of low-flow malformations. Hypointense areas are caused by thromboses or phleboliths^10^. Fluid–fluid levels can be attributed to hemorrhage or elevated protein content^11^. Gadolinium enhancement in venous malformations is slow and gradual in comparison to arterio-venous malformations, which exhibit early and fast filling with a contrast agent^11^. In macrocystic lymphatic malformations, Gadolinium enhancement can be observed only at the rim and the septa^2,12^, whereas microcystic lymphatic malformations usually show no significant enhancement^2^. Capillary malformations occur at skin level. Thus, only a thickening of skin or subcutaneous tissue can occasionally be observed in MR images^2,13^.

For an anatomical description, we refer to an anatomic framework proposed by Prasad et al. (2016) to analyze nerve involvement^9^.

## Results

Out of 299 patients (204 females; mean age 23.8 ± 18.2 years) with a simple or combined VM of the pelvis and/or thighs, we found 27 (9.03%) with an involvement of the sciatic, peroneal and/or tibial nerve. Three cases were excluded due to insufficient image quality caused by motion artefacts. One case was excluded because the MR protocol did not encompass all required sequences. 23 individuals (7.7%) (10 females; mean age 22.3 ± 12.2 years) met our previously defined inclusion criteria. Of these 23 patients (Table 1), 4 (17.4%) had a bilateral and 19 (82.6%) a unilateral (9 left- and 10 right-sided) VM. The study population encompassed 17 adults (mean age 27.8 years), and 6 children (< 18 years; mean age: 6.5 years). Among eight patients with simple VMs, one individual was diagnosed with PTEN-Hamartoma-Tumor syndrome and another one with a Cutaneomucosal Venous Malformation (VMCM). Combined VMs were identified in 15 patients. Within the combined VM group of patients, nine had capillary-lymphatic-venous malformations (CLVMs), which included one patient with CLOVES-syndrome and another with Klippel-Trénaunay-syndrome.

**Table 1:** Information per patient.

Pat. No	Affected nerve sections (Fig. 1)	Affected side	Clinical classification	MRI diagnosis at nerve, nerve infiltration pattern^9^	Muscle involvement	Nerve cross-sectional ratio ${\varvec{q}}$ of all affected sections (median)	Pain (VAS)	Restriction in motion
1	1 to 5	b	CVM	VM, SE	B		1	Mild
2	1, 2	l	VM	VM, SE	B	2.84	7	Mild
3	1 to 3	l	CLVM	VM, SP	B	5.63	N/A	N/A
4	1 to 5	r	CLVM	VM, SP-SE	B	3.76	5	Mild
5	1	l	VM	VM, SE	G	4.37	8	Severe
6	2 to 5	l	CLVM	VM, EN-SP	B	3.59	9	Severe
7	3, 4	r	CVM	VM, SE	B	3.74	1	Mild
8	1 to 4	l	VM	VM, SE	B	3.95	2	Mild
9	2 to 4	r	CLVM	VM, SP-SE	B	3.14	N/A	N/A
10	1, 2	b	VM	VM, SP	B		3	Mild
11	2, 3	r	LVM	VM, SE	B	2.99	4	Mild
12	3, 4	b	CLVM	VM, EN-SP	B		7	Severe
13	1 to 5	r	VM	VM, SP	B	4.91	8	Mild
14	1 to 5	l	CLVM	VM, SP	B	4.60	7	Mild
15	1 to 4	r	CLVM	VM, SP	B	3.60	3	Mild
16	1, 2	l	VM	VM, SE	G	9.10	N/A	N/A
17	1, 2	b	VM	VM, SE	B		7	Mild
18	1, 2	r	CLVM	VM, SP-SE	B	4.07	8	Severe
19	1 to 3	r	VM	VM, SE	H	8.25	7	Severe
20	1 to 3	r	LVM	VM, SE	B	10.16	8	Severe
21	1	l	CVM	VM, SP	H	4.08	6	Mild
22	1 to 3	l	LVM	VM, SE	H	3.17	3	Mild
23	1, 2	r	CLVM	VM, SE	B	4.90	N/A	Severe

The nerve cross-sectional ratio *q* is only calculated for patients with unilateral VM manifestation.

Affected nerve section refers to the predefined landmarks, as depicted in Fig. 1.

Affected side: *b* both, *l* left, *r* right.

Muscle involvement: *B* both gluteal and hamstring muscles, *G* gluteal muscles only, *H* hamstring muscles only.

Nerve infiltration pattern following the nomenclature of Prasad et al.^9^: *SE* subepineurial, *SP* subparaneurial, *EN* extraneural.

*VAS* visual analog scale. *N/A* not available.

None of the patients included in the study had undergone previous interventions or surgery of the sciatic nerve.

20 of the 23 patients completed the standardized disease-specific questionnaire. 19 patients provided pain responses, resulting in a median pain level of 7 (Range 1–9) on a 0-to-10 visual analog scale. 20 patients indicated that they experienced restrictions in motion to varying degrees: Mild limitations were reported by 13 patients, mainly occurring under heavy exertion. The other seven patients reported severe restrictions in motion occurring even during light daily activities.

In the MR images across all patients, an intraneural manifestation of the malformation was observed in at least one of the defined landmarks. The median ratio of the nerve’s cross-sectional areas on the involved side compared to the noninvolved side was $q$ > 1 at all measured points among the 19 patients with a unilateral manifestation. A cross-sectional area ratio of $q$ > 1 implies that the affected nerves presented enlarged in MR images (Table 2).

**Table 2:** List of cross-sectional areas of affected and healthy limbs in 19 patients with unilateral manifestation, providing the median cross-sectional area and the median ratios q of affected and unaffected side.

Landmark	n	Cross-sectional area A: Median (in cm^2^)		Ratio ${\varvec{q}}$: Median (25th; 75th percentile)
		Affected side	Unaffected side	
**(1) Sciatic nerve: proximal**	**15**	**1.33**	**0.33**	**4.08 (2.96; 6.8)**
Extraneural	2	1.08	0.8	
Subparaneurial	7	1.32	0.36	3.76 (2.0; 6.14)
Subepineurial	9	1.35	0.36	3.37 (2.97; 8.11)
**(2) Sciatic nerve: intermediate**	**16**	**0.99**	**0.25**	**3.77 (2.79; 5.78)**
Extraneural	1	[0.83]	[0.1]	[8.51]
Subparaneurial	8	0.83	0.17	3.77 (3.73; 5.08)
Subepineurial	9	1.33	0.28	3.25 (2.45; 6.45)
**(3) Sciatic nerve: division**	**12**	**0.88**	**0.22**	**3.89 (3.09; 5.73)**
Extraneural	1	[0.57]	[0.15]	[3.89]
Sub-paraneurial	5	0.91	0.22	3.97 (3.61; 5.82)
Subepineurial	7	0.84	0.25	3.17 (2.94; 5.23)
**(4) Peroneal nerve**	**7**	**0.30**	**0.08**	**3.94 (2.86; 4.82)**
Extraneural	1	0.15	0.07	[2.08]
Subparaneurial	5	0.3	0.09	3.89 (3.05; 4.46)
Subepineurial	4	0.23	0.07	4.55 (4.0; 4.92)
**(5) Tibial nerve**	**5**	**0.45**	**0.16**	**3.29 (2.64; 4.49)**
Extraneural	1	0.3	0.09	[3.29]
Subparaneurial	3	0.53	0.16	3.55 (2.34; 4.79)
Subepineurial	4	0.27	0.11	[3.88]

Values are given for each pre-defined landmark and broken down by affected anatomical compartments according to^9^. Note that VMs can manifest in more than one anatomical compartment of the nerve. Numbers in square brackets indicate that only 1 patient contributed.

**Figure 3:**
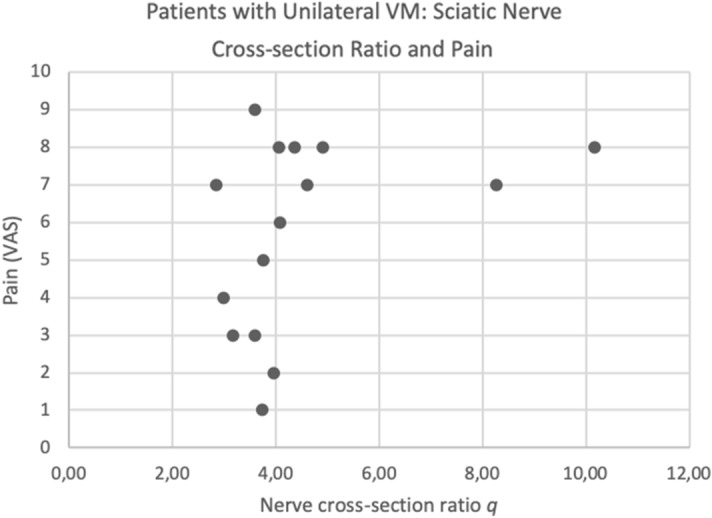
Association of cross-sectional area ratio q and pain level. *VAS* Visual analog scale.

An association of the cross-sectional area ratio $\mathrm{q}$ and the reported pain level (Fig. 3) was found.

**Figure 4:**
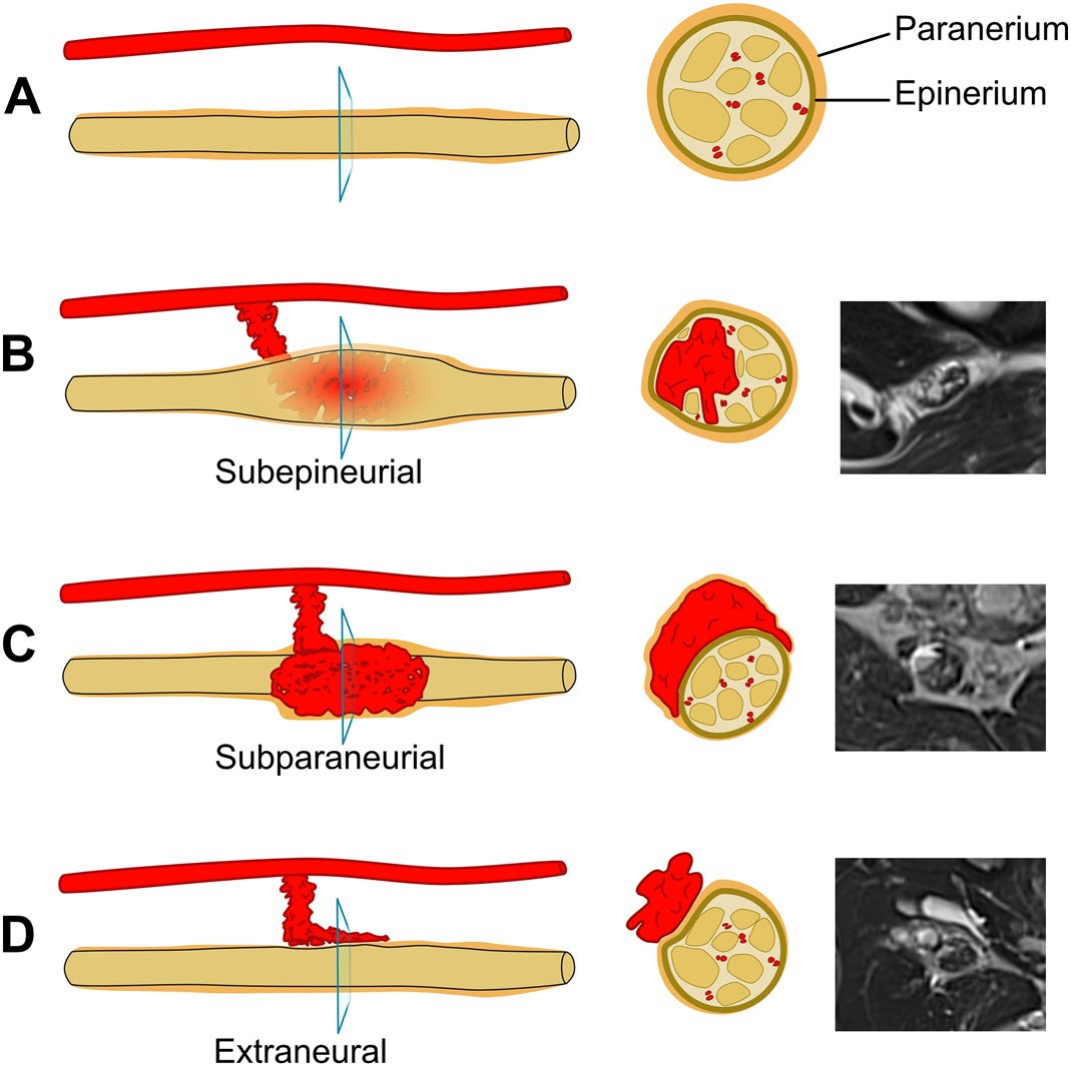
Infiltration patterns of vascular anomalies in neural tissue following the classification of Prasad et al.^9^. The left and middle row show schematic illustrations of infiltration patterns in longitudinal and cross-sectional view, respectively. The right row exemplarily displays the corresponding infiltration patterns observed on axial T2-weighted images. (**A**) Normal nerve without any infiltration by a vascular anomaly. (**B**) VM with subepineurial infiltration of the sciatic nerve. (**C**) VM with subparaneurial infiltration of the tibial nerve. (**D**) VM with extraneural manifestation around the sciatic nerve.

Dilated intraneural vessels were clearly detected in the T2-weighted (Fig. 4) and T1-weighted Gadolinium enhanced sequences in 19 of the 23 patients (82.6%). In all 23 patients, the local findings showed strong hyperintensity of the affected sciatic, peroneal or tibial nerves in fat-saturated T2-weighted sequences. In patients with combined slow-flow malformations, we found that only the venous portions infiltrated the sciatic nerve. Lymphatic or capillary portions of these malformations, when present, did not involve the affected nerves in any patient. Capillary portions of combined malformations, which were found in physical examination, occurred only at the skin level and therefore, did not involve structures of the sciatic nerve.

We describe the intraneural infiltration patterns following the anatomical framework proposed by Prasad et al.^9^ (Fig. 4). In line with their classification system, 2 of our patients (8.6%) showed combined extraneural-subparaneurial, 3 (13.04%) combined subparaneurial-subepineurial, 6 (26.1%) subparaneurial and 12 (52.2%) subepineurial infiltration patterns of the sciatic, peroneal and/or tibial nerves. Across all of the aforementioned landmarks of the sciatic and tibial nerves, subepineurial infiltrations were predominantly observed. The peroneal nerve (landmark 4) was the exception, with mainly subparaneurial infiltration patterns (for a detailed list, see Table 2). The paraneurium (Fig. 4C) is the tissue that allows nerve motility and establishes a connection to the structures surrounding the nerve^14^.

**Figure 5:**
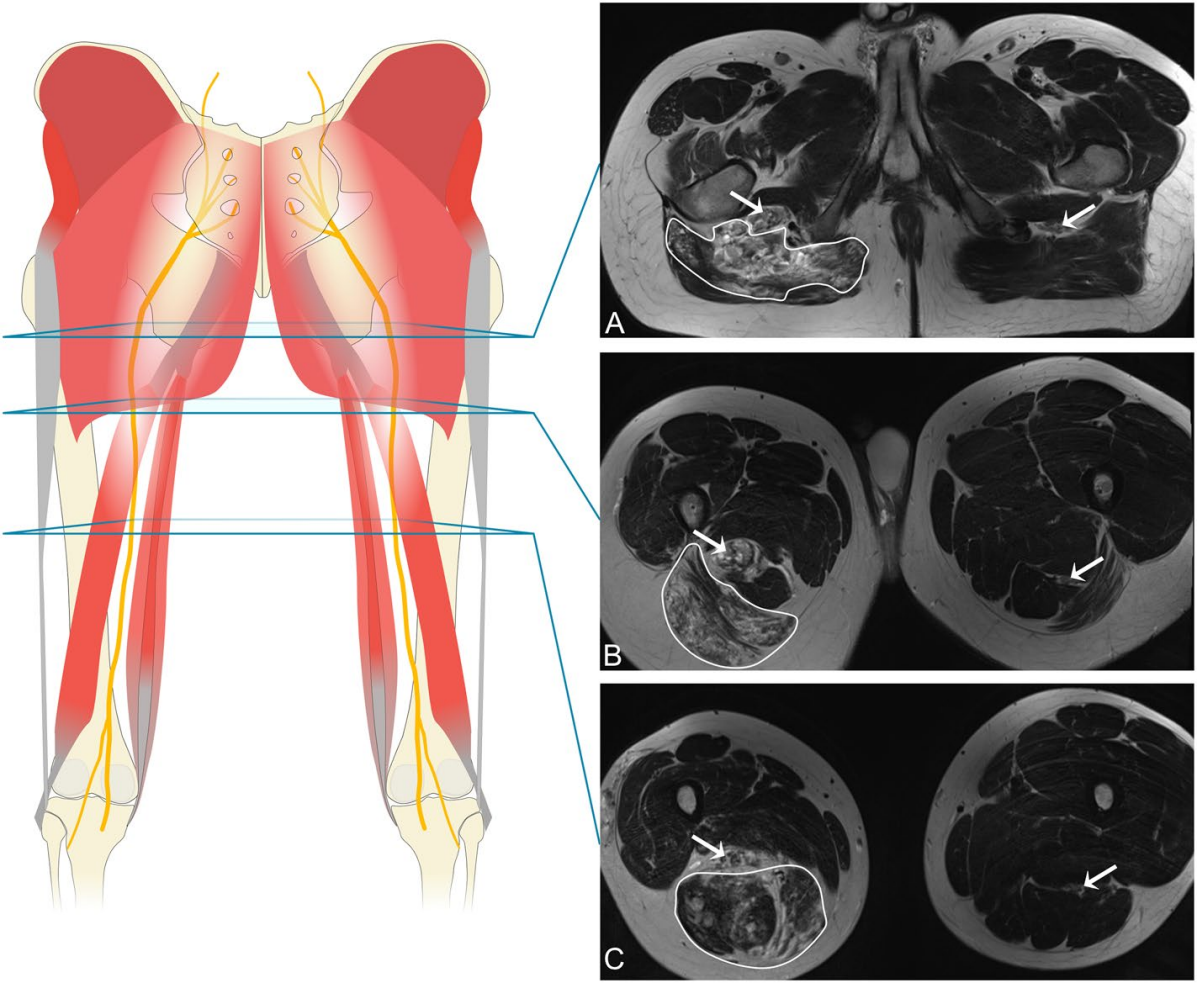
Widespread venous malformation of the right thigh infiltrating into gluteal and hamstring muscles and the sciatic nerve. (Patient 20). The sciatic nerve is marked with an arrow on the affected and healthy side. Outlined areas signify infiltrated muscles. (**A**) All gluteal muscles infiltrated by the VM. (**B**) Distal portion of gluteus maximus and proximal hamstrings infiltrated by the VM. (**C**) Hamstring muscles (semimembranosus, semitendinosus, biceps femoris) infiltrated by the VM.

**Figure 6:**
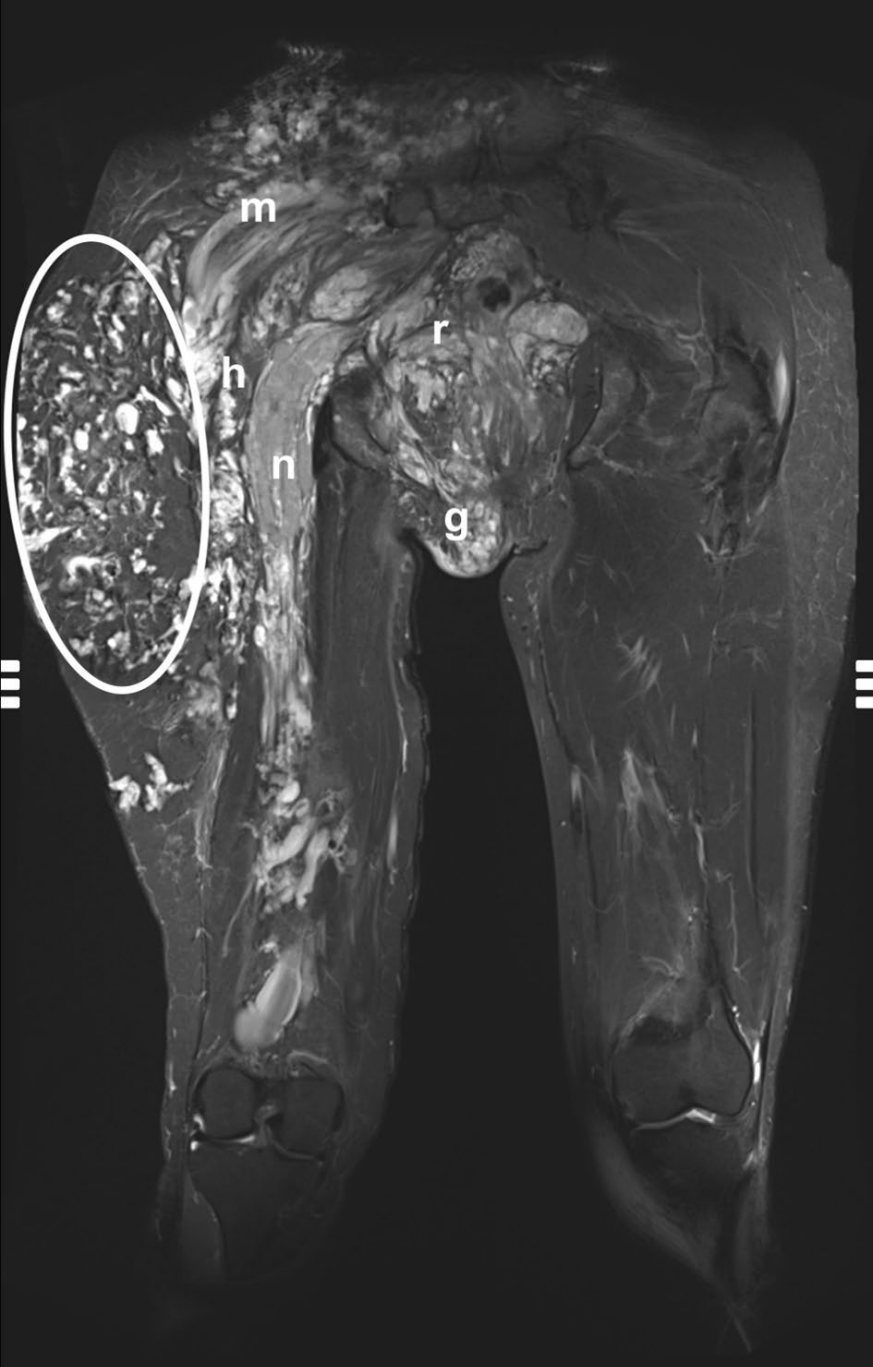
Extensive venous malformation in a 39-year old female patient. The composed T2-weighted STIR image in the coronal plane shows a subparaneurial involvement of the sciatic nerve (n), the gluteal (m) and hamstring (h) muscles, the rectum (r), the genital area (g) and the subcutis of the right thigh (ellipse).

With respect to the muscles surrounding the sciatic nerve (Fig. 5), we found certain muscles groups to be affected by the VM (Table 1). In all 23 patients, gluteal and/or hamstring muscles were involved: 18 patients (78.2%) had both gluteal and hamstring involvement, 3 (13%) patients had exclusive involvement of the hamstring muscles and 2 (8.7%) individuals had sole involvement of the gluteal muscles. In 12 of 12 cases (100%) of extensive VMs (VM visible at more than 3 landmarks) infiltrating the nerve, both the gluteal and hamstring muscles were involved in the VM (Fig. 6). The hamstring muscles (biceps femoris, semimembranosus and semitendinosus) were involved in 21 of 21 patients (100%) with a VM in intermediate and distal parts of the sciatic nerve (landmark 2 to 5). The gluteal muscles were affected in 15 of 18 patients (83.3%) with a proximal sciatic VM (landmark 1).

In one case (Patient No. 21), a proximal infiltration (landmark 1) of the sciatic nerve was diagnosed without accompanying involvement of the gluteal muscles, but the VM was present in the subcutaneous fat and connective tissue.

## Discussion

This data suggests an association of high clinical relevance: Sciatic nerve involvement consistently co-occurs with extensive intramuscular manifestations of venous malformations in hamstring and/or gluteal muscles. We assume that the knowledge of this association can facilitate the assessment of lower extremity VMs in regards to nerve involvement. Taking a closer look at the neural structures, the enlargement of the affected nerve in comparison to the healthy side provides a highly consistent pattern suitable for detecting nerve involvement. The accompanying hyperintensity of the neural tissue and its surrounding structures in T2-weighted images can be regularly observed and interpreted as sign of irritation and inflammation.

The prevalence of VMs in lower extremity muscles has been reported by Hein et al.^15^. Among 46 individuals, they found 6 patients (13%) with the gluteal muscles and 9 patients (19%) with the hamstring muscles involved in simple VMs. In their collective, they reported less involvement of these muscle groups in comparison to our results. The difference can be explained by our selection of cases with nerve involvement and the inclusion of not only simple, but also combined VMs and syndromes.

Van Gompel et al.^8^ presented a case series of vascular malformations (2 of 4 patients with a VM) causing sciatic neuropathy. Similar to our findings, their MR examinations showed enlarged and hyperintense sciatic nerves in T2-weighted images. In one patient, at the 6-month follow up after external and internal neurolysis, the nerve showed decreased hyperintensity in T2^8^. Examining the provided images of this previous work, within the limitations of image quality, we categorized their finding as a subparaneurial venous malformation.

A radiological classification system based on an anatomic framework was proposed by Prasad et al.^9^ with the intent to facilitate surgical decision-making. While presented for nine patients with intraneural lesions, their system has also proven applicable to our patient cohort. Our findings strongly confirm their statement that subepineurial manifestations of VMs are accompanied by nerve enlargement and signal hyperintensity in T2-weighted images^9^. Furthermore, we assume that the classification by Prasad et al. is not limited to the surgical decision-making process and can also be applied to planning and follow-up of conservative or interventional treatment.

We are well aware of the limitations of our study. There was no histological confirmation of the diagnosis. However, since physical examination, diagnostic ultrasound and MRI guide the treatment of VMs^13,16^, we decided not to conduct biopsies in our study. MRI is well established in the diagnosis and long-term management of VMs, with a sensitivity of 98.9% and a specificity of 90% that are comparable to Phlebography (sensitivity 97.3%, specificity 91.7%)^17^. MR imaging can show the dimensions of a VM and the involvement of nearby structures and additionally categorize VMs and differentiate them from other malformations^6^. The excellent contrast and resolution of 3 T MR neurography allows the appraisal of fascicular patterns and perineural structures^18^. Still, imaging and interpretation pitfalls should be kept in mind throughout the diagnostic process. T2 hyperintensity and the difference in thickness of the affected nerve have to be verified by comparison to the healthy side in order to rule out false-positives^19,20^. This limitation has to be kept in mind in the examination of bilateral manifestations of VMs. However, in our cohort, unilateral manifestations of VMs in the lower extremity occur more often than bilateral manifestations. Therefore, we assume that the consequences of this interpretation pitfall are marginal. Neurological diagnostic data (i.e. electroneurography) were not collected in our patient analysis. Instead, we followed clinical symptoms using a standardized assessment of pain and restriction in motion. In this context it should be mentioned that nerve lesions can be detected and localized in MR imaging with high spatial resolution^21^ at an early stage before changes appear in electroneurography or electromyography^22^. Therefore, we advise that MRI findings of nerve involvement are an indication for additional neurological examination to prevent aggravation of pain or neurological deficits. Since multiple case reports indicate that nerve-associated vascular anomalies may have an effect on electromyographic findings^8,23^, we anticipate that further research in this field is necessary.

## Conclusion

This study provides a clinically applicable and highly relevant approach to the MRI-based assessment of simple and combined venous malformations (VMs) in the lower extremity. We strongly recommend all clinicians examining MR images of venous malformations to be vigilant about the possible involvement of nerve structures. The highly consistent pattern of involvement of the gluteal and hamstring muscles should be used to guide the radiologist to a closer examination of the nerve structures. Muscle involvement, differences in nerve diameter, hyperintensity of the nerve in fat-saturated T2-sequences and dilated intraneural vessel structures are indicators of nerve infiltration. Consequently, intraneural findings in MR imaging can influence decisions towards further neurological diagnosis and may even determine the feasibility of interventions. They also may call for an increased frequency of follow-up examinations (e.g. every six months) in the long-term therapeutic relationship. We believe that the observation of the nerve’s morphology in MRI should be part of any conservative management or interventional treatment of venous malformations affecting the lower extremity. Meticulous diagnosis and follow-up require a comprehensive framework for the morphologic description and measurements, as presented in this study.

## Abbreviations

VM: Venous malformation
ISSVA: International society for the study of vascular anomalies
VMCM: Cutaneomucosal venous malformation
CLVM: Capillary-lymphatic-venous malformation
STIR: Short tau inversion recovery
TSE: Turbo spin echo
TWIST: Time-resolved angiography with interleaved stochastic trajectories
